# Beyond physical ability—predicting women’s football performance from psychological factors

**DOI:** 10.3389/fpsyg.2023.1146372

**Published:** 2023-03-28

**Authors:** Susann Dahl Pettersen, Monica Martinussen, Bjørn Helge Handegård, Lene-Mari Potulski Rasmussen, Roman Koposov, Frode Adolfsen

**Affiliations:** Regional Centre for Child and Youth Mental Health and Child Welfare, UiT-The Arctic University of Norway, Tromsø, Norway

**Keywords:** soccer, sports psychology, personality, motivational climate, team sport, football, performance

## Abstract

**Introduction:**

Even though there is a clear agreement among researchers that psychological factors are a vital part of a football player’s performance, the topic has not been investigated thoroughly. The present study aimed to examine the predictive value of psychological factors on female football players’ match performance.

**Methods:**

A sample of 156 players from the top two leagues in Norway completed the following questionnaires: Perceived Motivational Climate in Sport Questionnaire 2 (PMCSQ-2), Big Five Inventory (BFI-20), Self-Regulated Learning questionnaire, and Grit-S and Sport Mental Toughness Questionnaire (SMTQ). Match performance data were collected from the online database of the performance analysis company InStat.

**Results:**

Results from a linear mixed model analysis showed that perceived mastery climate and extraversion were the only significant predictors of performance. Other relevant indicators, such as mental toughness, self-regulated learning, and grit, did not predict performance.

**Discussion:**

These findings suggest that the team climate facilitated by coaches may be more important for predicting match performance than individual psychological factors.

## 1. Introduction

Football (soccer) is played worldwide by men and women of all ages and all skill levels. In terms of match attendance, media attention, salaries, and sponsorship, high-level and elite female football has lagged far behind that of their male counterparts (FIFPRO, 2020). During the past 20 years, however, there have been positive developments in most of these areas, in addition to a growing body of research concerning female players (Kirkendall, 2020), although this is not comparable to the amount of research conducted on male football players (Kryger et al., 2021; Alves et al., 2022). The most prevalent research areas in the field of women’s football have been related to sports medicine, followed by physiology and sociology (society, patterns of social relationships, social interaction, and culture; Kryger et al., 2021). Psychological factors have received less attention, although there is a clear consensus regarding the importance of mental strength to athletes’ performance (Fletcher and Sarkar, 2012; Herrero et al., 2021; Sillero et al., 2021). A recent systematic review found that there were only 14 studies examining the relationship between performance and psychological factors in female football players (Pettersen et al., 2021), while a review article identified only six articles that investigated sport psychology in elite-level female soccer players (Kirkendall and Krustrup, 2021).

Although psychological factors have been deemed to be important for football performance, most studies focus on physical, tactical, and technical aspects of the game, and measurements of performance vary greatly between the studies. The measurement of football performance has traditionally focused on technical execution performance, such as shooting, passing, goals, and assists (Yi et al., 2020), as well as physiological parameters, such as sprint distances, total distances covered, accelerations, and peak speed in matches and training sessions (Krustrup et al., 2005; Baptista et al., 2022). However, these measurements do not take into consideration the full complexity of football performance, which also entails position-specific tactical performance. For instance, when using the number of goals and assists as a performance outcome, the goalkeeper and the defenders will inevitably fall short, compared to the midfielders and forwards. Furthermore, the total distance covered in a match might tell us something about the endurance capacity of a player, but it cannot show whether a player performed the runs in the most efficient ways to, e.g., create goal-scoring opportunities or stop the opposing team from creating goal-scoring opportunities. With the emergence of companies/systems analyzing performance, such as InStat, Stats Perform, and Wyscout, new ways of objectively measuring individual performance have become available (Castellano et al., 2014). For instance, InStat (2023) provides position-specific data on tackles won, air challenges, ball recoveries in opponent’s half, accurate crosses, key passes, lost balls, and successful dribbles. The development of performance analysis tools has made studies of psychological factors and how they affect performance more optimal, yielding more valid results.

Previous studies of psychological factors and football performance have often distinguished between measurements of teams and of individuals. Outcome measures of performance at team level are often rated by comparing different team levels, successful and unsuccessful teams, or goals and assists. The variability of outcome measures and the appropriateness of these have been questioned in recent literature (Ivarsson et al., 2020), and a well-developed football performance measure has been called for by several researchers (Piedmont et al., 1999; Vestberg et al., 2012; Ivarsson et al., 2020). Other studies have used subjective performance measures, i.e., the players have rated their own performance in matches (Olmedilla et al., 2019). Subjective measures may, however, have a high risk of bias, and the reliability and construct validity are often unknown (McGrath, 2010; Ivarsson et al., 2020). Considering the scarcity and variability of football performance measures, it is challenging to compare and generalize previous findings. Nevertheless, the findings may provide indications as to which factors are most important for football performance.

A recent systematic review showed that higher-level female football players had elevated scores for some psychological factors (Pettersen et al., 2021). For example, grit (passion and perseverance for long-term goals; Duckworth et al., 2007) showed a tendency to increase with the increasing level of the players (Meyer et al., 2017; Sigmundsson et al., 2020a), as did mental toughness (the ability to achieve personal goals in the face of pressure from a wide range of different stressors; Hardy et al., 2014) in both female (Danielsen et al., 2017) and male (Guillen and Santana, 2018) football players. The personality trait of conscientiousness has also been associated with better football performance when performance is rated by coaches (Piedmont et al., 1999). A further personality trait, extraversion, has also been associated with performance, in the form of champion athletes having higher scores for this trait than non-champion athletes, regardless of the type of sport (Piepiora, 2021). Conscientiousness can be defined a tendency to be organized and dependable, show self-discipline, aim for achievement, and prefer planned behavior (John and Srivastava, 1999), while extraversion can be defined as a personality trait characterized by outgoingness, social dominance, and a tendency to seek stimulation and excitement in social situations (Costa and McCrae, 2008).

Few articles have examined the relationship between the perceived motivational climate and football performance directly, but some studies have been conducted using indirect performance measures. A perceived motivational climate refers to the situational and environmental cues that are emphasized, a coach regarding what is important, valued, and expected (Duda, 2001). For instance, one study showed that female players who placed emphasis on a perceived mastery climate, along with positive and informative feedback from a coach, reported greater perceived football competence, enjoyment, and intrinsic motivation in football (Weiss et al., 2009). A similar study showed that mastery climate (i.e., task involvement) and task orientation were positively associated with practice strategy use, peaking under pressure and mindful engagement in high school female football players (Iwasaki, 2015). Additionally, as with other psychological factors, scores for self-regulated learning (planning, evaluating and reflecting upon sports development) have been shown to increase with increasing performance level and experience in football players (Toering et al., 2009, 2012). However, to the best of our knowledge, no studies have used psychological factors as predictors of objective performance measures in high-level and elite women’s football.

With previous findings in mind, we aimed to combine a comprehensive number of psychological factors to predict individual objective match performance. Although these factors have been examined separately before, no study has combined them and used an objective measurement tool (such as the InStat Index) to measure match performance. We hypothesized that mental toughness, grit, extraversion, conscientiousness, perceived motivational climate, and self-regulated learning would predict individual objective match performance in female football players (Piedmont et al., 1999; Toering et al., 2009; Weiss et al., 2009; Danielsen et al., 2017; Meyer et al., 2017).

## 2. Method

### 2.1. Participants

The sample consisted of 156 participants, all of whom were female football players from the top (*n* = 57) and second (*n* = 99) leagues in Norway. The participants had an age range between 16 and 30 years (*M* = 21.43, SD = 3.40). The athletes were recruited from 17 of the top 20 football clubs in Norway—nine from the highest level, and eight from the second level. One team declined participation, while two of the teams did not respond to our inquiries. Demographic variables, such as age, football team, training hours, injuries, mental training, years playing organized football, and league level, were all collected through questionnaires (Table 1). In addition, we coded whether the football matches were played at home or away, to control for home advantage in the analyzes.

**Table 1:** Demographic characteristics of the participants (*N* = 156).

Demographic characteristics	*n* (%)
**Work**	
Full-time	14 (9.0)
Part-time	55 (35.3)
**Study**	
Full-time	69 (44.2)
Part-time	12 (7.7)
**Football career**	
Playing in a national team	26 (16.7)
Previously played in a national team	79 (50.6)
**Type of contract**	
Professional	66 (42.3)
Amateur	89 (57.1)
**Position**	
Goalkeeper	21 (13.5)
Defense	45 (28.8)
Midfield	45 (28.8)
Forward	45 (28.8)
**Mental training**	
Never	82 (52.6)
Monthly	50 (32.1)
Weekly	17 (10.9)
Several times a week	7 (4.5)
**Injury**	
Been injured in the past year	76 (48.7)
**Injury duration**	
Two weeks	7 (4.5)
One month	14 (9.0)
Two months	17 (10.9)
Three months	8 (5.1)
Over three months	30 (19.2)

The number of responses per team varied, with a mean of 9.18 (range 3–21, SD = 4.75). Participants were all part of team squads in the 2021 season, training on average 7.05 (SD = 1.69) hours with the team, and 3.63 (SD = 2.40) hours on their own, per week. Approximately half of the players had never practiced any mental training (52.6%). About half of the players were studying (51.9%) and 44.3% of the players were working in addition to playing football (Table 1). A total of 35.2% of the players reported having been injured for 2 months or more during the previous year.

### 2.2. Instruments

BFI-20 was used to measure personality traits. BFI-20 is a short scale version of the Big Five Inventory, which in its original form consists of 44 items (Engvik and Clausen, 2011). BFI-20 consists of five subscales (Openness, Conscientiousness, Extraversion, Agreeableness, and Neuroticism), and each subscale consists of 4 items. Items were rated on a 7-point Likert scale (1 = does not fit, 7 = fits perfectly). As openness, agreeableness, and neuroticism have not been associated with football performance in previous literature, they were not included in the analysis. An example item of extraversion is, “I am someone who is outgoing, sociable,” while an example item of conscientiousness is, “I am some who does a thorough job.” Cronbach’s alpha values for each subscale for the current study were: Conscientiousness (*α* = 0.63) and Extraversion (*α* = 0.85).

Grit-S was used to measure grit, which is regarded as a non-cognitive personal quality, and is defined as perseverance and passion for the achievement of long-term goals (Duckworth et al., 2007). The instrument consists of eight items, rated on a 5-point Likert scale (1 = not at all like me, 5 = very much like me). An example item is, “I finish whatever I begin.” Cronbach’s alpha for the full scale for the current study was *α* = 0.75.

Sport Mental Toughness Questionnaire (SMTQ) was used to measure mental toughness (Sheard et al., 2009). This questionnaire has 12 items and consists of three subscales: confidence, constancy, and control. Items were rated on a 4-point Likert scale (1 = not true at all, 4 = very true). An example item for confidence is, “I have an unshakeable confidence in my ability,” an example item for constancy is, “I take responsibility for setting myself challenging targets,” while an example of a reversed item for control is, “I worry about performing poorly.” Cronbach’s alpha values for each subscale for the current study were: Confidence (*α* = 0.75), Constancy (*α* = 0.64), and Control (*α* = 0.68).

Perceived Motivational Climate in Sport Questionnaire-2 (PMCSQ-2) was used to measure the perceived mastery climate (Newton et al., 2000). PMCSQ-2 consists of two subscales: ego-climate and mastery climate. As ego climate have not been associated with football performance in previous literature, it was not included in the analysis. The subscale of mastery climate consists of 17 items and items were rated on a 5-point Likert scale (1 = strongly disagree, 5 = strongly agree). An example item for mastery climate is, “My coach encouraged players to help each other learn.” Cronbach’s alpha for the subscale for the current study was: Mastery climate (*α* = 0.93).

Self-regulated learning was measured using a football-specific instrument, as described in Toering et al. (2013). The questionnaire consists of 22 items, divided into three subscales: Evaluation, Reflection, and Planning. Items were rated on a 5-point Likert scale (1 = never, 5 = always). An example item for evaluation is, “Each practice session I think back and evaluate whether I did the right things to become a better player,” an example item for reflection is, “During each practice session I check what I still have to do to reach my practice goal,” while an example item for planning is, “Before each practice session I plan which skills I want to work on during the session.” Cronbach’s alpha values for each subscale for the current study were: Evaluation (*α* = 0.88), Reflection (*α* = 0.90), and Planning (*α* = 0.82).

#### 2.2.1. Football performance measure

Objective individual performance was measured using InStat, which provides a detailed objective performance analysis of football matches and yields a position-specific index (InStat Index) (InStat, 2016). The index is a result of action coefficients, such as passes, dribbles, and shots multiplied by a weighted match level coefficient (WMLC). A total of 12–14 key parameters are used to assess action/player performance, depending on the player’s position. For example, forwards will be assessed on the percentage of offensive challenges won, while full backs will be assessed on defensive challenges won. The WMLC is automatically calculated based on the quality of the actions of a player, the quality of teammates’ actions, and the opponent’s level (earlier match indexes). The better the quality of a match, the higher the WMLC value will become. Players must spend a minimum amount of time on the field and perform a minimum number of actions for the index to be calculated. A team index is also made available in the match report, comprising all the players’ average indexes. The InStat Index assures statistical accuracy by means of a multi-level system of verification performed by managers, supervisors, and inspectors, in addition to strict implementation of their methodology in the match analysis. The InStat Index has been used as a match performance measure in related research (Modric et al., 2019; Reneker et al., 2020), and has been shown to have high inter-operator reliability (Silva and Marcelino, 2022).

**Figure 1:**
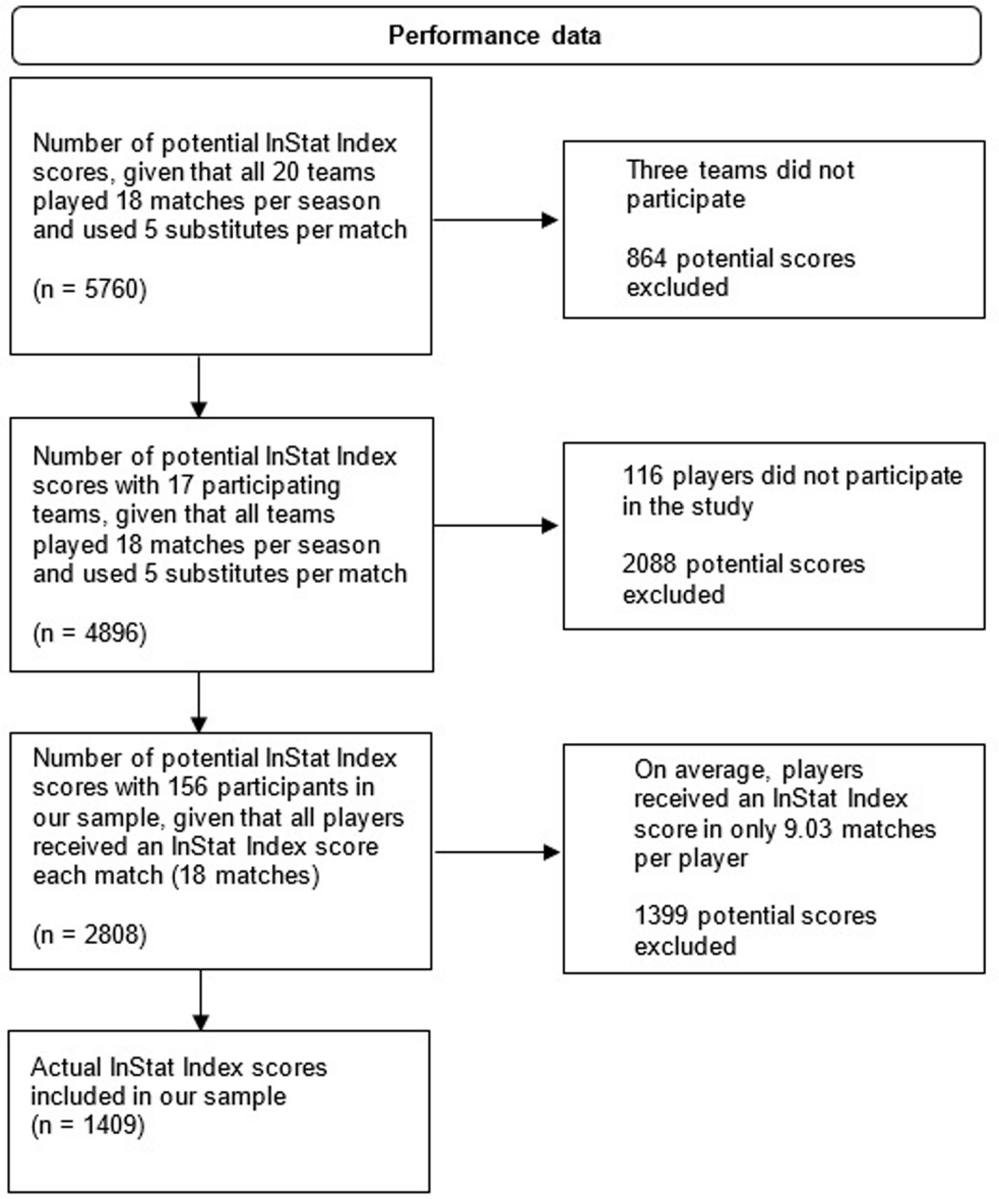
Flow diagram of InStat Index scores.

As there were no official player database providing information about how many players were registered in the two top leagues, the potential number of InStat scores we could obtain (5760) is based on the maximum theoretical number of matches (see Figure 1). This includes the assumption that all 20 teams played 18 matches and used five substitutes in each match. With 156 players participating in the study, there were 2,808 potential matches to be rated, but as players only had an InStat score in 9.03 matches on average, the total number ended up being 1,409 (50.2%). The reasons why some players did not obtain an InStat score are threefold: the player did not participate in the match; the player came on as a substitute, but did not play for long enough or did not perform enough actions to get a score; video systems were not available at the stadium.

### 2.3. Procedure

All teams in the top and second leagues in Norway were contacted *via* an email sent to the club’s sports director or manager with information about the study. All teams were also offered a presentation of the project. Only three meetings with a presentation of the project were conducted physically, and the remaining 11 meetings were held digitally (on Zoom, Skype or Microsoft Teams) as a result of restrictions due to the COVID-19 pandemic. The three remaining teams declined a presentation but received written information about the study. After the presentation, players were encouraged follow a link to answer the questionnaire. The link was sent to the team’s representative contact, which then distributed it to the players. The questionnaire was created on the webpage Nettskjema which is a tool for online data collection. Participants signed an online written consent form, with the option to withdraw at any time. All the information was available in English and Norwegian. After 2 weeks, the club, represented by a contact person, received a reminder for the players to complete the questionnaire. A second reminder was sent after 1 month.

Teams were contacted before the start of the season, but due to the COVID-19 pandemic the data collection stretched throughout 2021, with the last team responding in November. The questionnaire containing demographic variables and psychological factors was only answered one time, while the InStat score was recorded after every match (maximum 18 matches per player, national cup matches not included). The project was deemed to be outside the jurisdiction of the Health Research Act by the Regional Ethics Committee North’s mandate. The project was approved by NSD—Norwegian Centre for Research Data (project 133,802). Data were collected electronically and stored at Services for Sensitive Data (SSD).

### 2.4. Statistical analysis

Pearson correlations were used to examine the associations between the variables in the study. A Spearman’s rank correlation was computed to examine the association between the InStat Index and the end-of-season league standings. Elevated placements are coded with low numbers (1st place is coded 1, 2nd place is coded 2, etc.,). Intraclass correlations (ICCs) were computed using linear mixed models (LMM) on an unconditional means model (Singer and Willett, 2003). A linear mixed model (LMM) analysis was used to investigate associations between performance and selected independent variables. Backward elimination was used to remove non-significant predictors. As performance data were nested within players (multiple matches for each player), and players were nested within teams, the performance data are hierarchical. This creates dependent performance scores within players and teams, and the chosen method is able to handle such multilevel data. As a measure of “explained” variance a pseudo *R*^2^ was computed for the observed InStat values and values predicted by the final model (Singer and Willett, 2003). All analyzes were conducted using SPSS v.28.0 (SPSS, Inc., Chicago, IL, United States). The level of significance was set to 0.05.

## 3. Results

### 3.1. Correlations

Table 2 shows correlations between the variables used in the study, except for home advantage, as this is not measured on the player level. The InStat variable is an average of the players’ available match indexes for the season. Planning, evaluating, and reflection are all subscales of the self-regulated learning questionnaire, which accounts for the large inter-correlations (planning and evaluation *r* = 0.70, *p* = < 0.01, planning and reflection *r* = 0.73, *p* = < 0.05, reflection and evaluation *r* = 80, *p* = < 0.01). There was also a strong correlation between grit, constancy (mental toughness), and conscientiousness (grit and constancy *r* = 0.66, *p* = < 0.01, grit and conscientiousness *r* = 64, *p* = < 0.01, conscientiousness and constancy *r* = 0.62, *p* = < 0.01).

**Table 2:** Bivariate correlations between variables in the study.

Variables	*M*	SD	1	2	3	4	5	6	7	8	9	10	11	12	13
**Covariates**															
1. Years played	14.53	3.75	1												
2. Level	0.37	0.48	0.13	1											
**Psychological factors**															
3. Grit	3.72	0.50	0.07	0.13	1										
4. Confidence	2.88	0.48	0.13	0.13	0.38^**^	1									
5. Constancy	3.28	0.52	0.03	0.16^*^	0.66^**^	0.54^**^	1								
6. Control	2.24	0.65	0.01	0.14	0.31^**^	0.46^**^	0.40^**^	1							
7. Mastery	4.10	0.68	−0.03	−0.01	0.01	0.07	0.01	0.08	1						
8. Reflection	3.28	0.71	−0.19^*^	0.16^*^	0.28^**^	0.26^**^	0.35^**^	0.04	0.15^*^	1					
9. Evaluation	3.69	0.74	−0.15	0.21^**^	0.30^**^	0.29^**^	0.41^**^	0.03	0.10	0.80^**^	1				
10. Planning	2.57	0.61	−0.17^*^	0.16^*^	0.38^**^	0.28^**^	0.47^**^	0.07	0.16^*^	0.73^*^	0.70^**^	1			
**Personality traits**															
11.Extraversion	14.59	3.65	0.27^**^	−0.00	0.11	0.32^**^	0.13	0.14	0.16^*^	0.69	0.01	0.06	1		
12.Conscientiousness	15.87	2.61	0.04	0.09	0.64^**^	0.25^**^	0.62^**^	0.13	−0.03	18^*^	0.20^*^	0.35^**^	−0.02	1	
**Performance measure**															
13. InStat^a^	138.93	63.94	0.19^*^	0.09	0.01	−0.01	0.02	0.02	0.05	−0.09	−0.03	−0.04	0.4	−0.01	1

Level is coded top league (1) or second league (0).

a InStat is the mean score of a player’s InStat Index throughout the season (maximum 18 matches).

^**^p < 0.01, ^*^p < 0.05 (two-tailed).

### 3.2. InStat and season standings

Because the InStat Index has not been extensively used in research before, we wanted to examine the correlation between the team’s average InStat Index and the team’s end of season ranking in the league. A Spearman’s rank correlation was computed, and we found a strong negative correlation between the mean team InStat Index and the end of season ranking in the top league (*n* = 9), *r* = − 0.91, *p* = 0.002. Another Spearman’s rank correlation was computed to assess the relationship between the mean team InStat Index and the end of season ranking in the second league (*n* = 8). There was a strong negative correlation between the two variables, *r* = − 0.95, *p* < 0.001. The correlation is negative because a low placement will give a high number (such as 8, 9 or 10). The high correlation supports the construct validity of the InStat Index (Smith, 2005).

### 3.3. Predicting football performance

To determine the strength of the relationship between grit, personality (extraversion and conscientiousness), mental toughness (confidence, constancy and control), self-regulated learning (planning, evaluation and reflection), mastery climate, and InStat performance, a linear mixed model analysis was conducted (Table 3). Backward elimination was used to remove non-significant predictors. The analysis was conducted using home/away advantage, number of years playing football and league as control variables. The ICC with no predictors in the model showed that 11.5% (*p* < 0.001) of the variance in performance was found between players within teams, and 39.1% (*p* = 0.010) of the variance in performance was found between teams. Also, 49.3% of the total variation was located on the match level. The final model revealed that mastery climate, extraversion, and the mental toughness confidence subscale were significant predictors of performance (Table 4). As a measure of effect size in the final model, the correlation between the observed InStat values and the model’s predicted values were computed. The results showed a large correlation (*r* = 0.74, *p* < 0.001). The correlation was squared to give an indication of “explained” variance (pseudo *R*^2^ = 0.54) as seen in Singer and Willett (2003). This provides a measure of the overall contribution of the specific set of predictors included in the model.

**Table 3:** Linear mixed model, full model for predicting football performance.

Fixed effect	Coefficient	SD	*t*	*p*
Years played	1.19	0.31	3.88	<0.001
Level	21.01	6.05	3.47	0.01
Advantage	2.49	1.05	2.38	0.02
Grit	−2.82	2.62	−1.08	0.28
Confidence	−5.76	2.91	−1.98	0.05
Constancy	1.64	3.37	0.49	0.63
Control	−0.60	1.73	−0.35	0.73
Mastery climate	7.30	1.90	3.85	<0.001
Reflection	−2.16	2.58	−0.84	0.41
Evaluation	−1.36	2.55	−0.60	0.60
Planning	4.65	2.68	1.74	0.09
Extraversion	0.61	0.29	2.08	0.05
Conscientiousness	0.25	0.54	0.46	0.65
**Random effects**	**Variance component**	**SD**	***z***	***p***
Residual	372.64	14.82	25.14	<0.001
Players	61.80	14.83	4.17	<0.001
Teams	124.31	59.90	2.08	0.04

Covariates: Level = top league (1) or second league (0), Advantage = home (1) or away (0) match, Years played = years played organized football. Predictors: Mastery Climate (Motivational Climate), Confidence, Constancy, Control (Mental Toughness), Extraversion, Conscientiousness (Personality), Planning, Evaluation and Reflection (self-regulated learning) and Grit.

**Table 4:** Linear mixed model, final model for predicting football performance.

Fixed effect	Coefficient	SD	*t*	*p*
Years played	1.14	0.29	3.90	<0.001
Level	21.48	5.99	3.59	<0.01
Advantage	2.48	1.04	2.40	0.02
Confidence	−5.21	2.20	−2.37	0.02
Mastery climate	6.96	1.79	3.90	<0.001
Extraversion	0.62	0.28	2.18	0.03
**Random effects**	**Variance component**	**SD**	***z***	***p***
Residual	369.96	14.64	25.28	<0.001
Players	59.73	13.93	4.29	<0.001
Teams	123.97	58.42	2.12	0.03

Covariates: Level = top league (1) or second league (0), Advantage = home (1) or away (0) match, Years played = years played organized football. Predictors: Mastery Climate, Confidence (Mental Toughness), Extraversion (Personality).

## 4. Discussion

The current study aimed to investigate whether psychological factors such as mental toughness, grit, extraversion, conscientiousness, perceived motivational climate, and self-regulated learning may predict individual objective match performance among female football players. Our findings demonstrated that, among several psychological factors, a perceived mastery motivational climate and extraversion were the only significant predictors of individual performance in women’s football. The confidence subscale of mental toughness revealed a negative relation to match performance, although we question the validity of this finding because it is contradictive of previous findings (Danielsen et al., 2017; Kristjánsdóttir et al., 2019). It is difficult to understand why lower levels of mental toughness should be beneficial to football performance, and this possibly spurious finding may be a result of the specific measure used or perhaps a statistical artifact. Further studies should examine this relationship before making conclusions about the predictive value of mental toughness. Grit, conscientiousness, and self-regulation were not significant predictors of match performance.

Our results suggest that a mastery motivational climate is a predictor for individual match performance. This finding is interesting, as a motivational climate is not an inherent psychological factor, but rather the perceived team environment that is facilitated by the coach (es). Although motivational climate has not been directly linked to football performance in previous literature, studies suggest that a mastery climate is associated with satisfaction of the need for competence, autonomy and relatedness (Alvarez et al., 2012), global self-esteem (Reinboth and Duda, 2004), and engagement in sport (Curran et al., 2015). A further study found that youth football players (15–17 years), both male and female, experienced their motivational climate as being significantly more ego-oriented than the coaches did (Møllerløkken et al., 2017). An ego-oriented climate is the opposite of a mastery climate, where the coach is more focused on punishment for errors, unequal recognition, and rivalry among members of a team (Murcia et al., 2008)—i.e., by comparing star players to less successful players. This suggests that coaches might have to devote more effort and focus on creating and upholding a mastery motivational climate to ensure an optimal environment for performance.

Our finding on the linkage between extraversion and football performance is in accordance with previous studies. An extensive review examining extraversion in sport concluded with a few interesting points, namely that athletes were more extraverted than non-athletes, and that team-sport athletes were more extraverted than athletes in individual sports (Allen et al., 2021). During a football season, teams experience high-pressure situations, both leading up to matches and during important, high-stake matches, and players will inevitably experience high levels of stress. Research indicates that extraverted athletes use more desirable coping strategies that focus on the source of stress and social support (Raglin, 2001), compared to introverted athletes. Also, players who exhibit high levels of extraversion may have better communication skills (Macht et al., 2014). This could impact their performance on the pitch, making them better at communicating with and orienting teammates of their positioning, and giving positive feedback. In turn, these factors could explain why the personality trait of extraversion predicts match performance in female football.

One unanticipated finding was that the mental toughness subscale of confidence was a negative predictor of performance. This outcome was surprising, as earlier studies have shown that scores on confidence increase in line with an increase in the level of the players (Danielsen et al., 2017; Kristjánsdóttir et al., 2019). There is a possibility that this is a result of a statistical anomaly, and it should therefore be interpreted with caution. Another possibility is that self-reported confidence measured on a single occasion during the season is not an ideal predictor of match performance, as self-confidence may fluctuate during the season (Carpentier and Mageau, 2016). Further studies are needed to explore the impact of confidence and performance in women’s football.

Another unanticipated finding was that grit, conscientiousness, and self-regulated learning did not predict performance. Previous studies conducted on the association between grit and self-regulated learning and performance have shown that scores increase with the athletes’ level and experience (Sigmundsson et al., 2020a,b; Toering et al., 2009, 2013). Possible explanations for this are that these psychological factors develop throughout the players’ careers or that they are inherent traits that enabled them to succeed, or a combination of the two, but this does not have a direct effect on performance. It might also be that this group of selected individuals are too homogenous, and that the variations in the scores are too small to make a statistical difference. It is also possible that there are other factors that contribute more to performance, such as stress (Pensgaard and Ursin, 1998), anxiety (Woodman and Hardy, 2003), and level of arousal (Arent and Landers, 2003).

Although grit, self-regulated learning, conscientiousness, and confidence did not predict performance in our sample, we argue that they cannot be disregarded as contributors to an athlete’s success. Research findings indicate that individual psychological factors play a role in specific parts of the match, for example when performing a free kick in a favorable position or in a penalty situation (Jordet et al., 2007; Arrondel et al., 2019). As the InStat Index is an aggregated score from all actions, it does not separate set pieces and open play situations. Previous research has also examined whether psychological factors can predict future football performance, and found that only a few factors were significant (Ivarsson et al., 2020). These factors were task orientation, task-oriented coping strategies and perceptual-cognitive functions. However, the effect sizes were small. The authors also described various biases in the studies included, and concluded that there was uncertainty around the level of scientific evidence for the precise role psychological factors have and how they affect future football performance. However, our sample had a higher level of grit compared to a similar sample of students (Sigmundsson et al., 2020b), and it is possible that individual psychological factors such as grit are important for performance, but that players with lower levels quit earlier in their career.

These findings, although preliminary, suggest that a perceived mastery climate and extraversion are important psychological factors for performance in women’s football. Despite these results, questions still remain. Further work should be undertaken to investigate the nature of psychological factors in relation to performance in football, for example to examine whether these factors develop through a player’s career or whether they are inherent traits that led to success. A recommendation for future research is to also measure the psychological factors before each match, to explore whether factors such as self-confidence change during a season and whether this affects match performance.

### 4.1. Limitations

Although previous studies have encouraged researchers to collect larger samples, the present study was limited by the available number of teams in the two highest female football leagues in Norway (20 teams). The relatively low number of teams available for this study creates power limitations. One thousand four hundred and nine observed performance scores may seem like a large number but considering the relatively high dependency in performance scores both at the player level and the team level, the effective sample size is substantially lower than this. In addition, all questionnaire data should have been collected before the start of the season, but this was also challenging due to the COVID-19 pandemic and a long response time from the teams. The prolonging of the data collection may have affected the results. Another limitation was that home or away advantage was the only predictor on match level. Since 49.3% of the total variation was located on match level, future studies should aim to include predictors that are measured before each match. This might include the wellness of the players, motivational factors, stress, readiness to play, etc. Furthermore, the football season was shortened due to the COVID-19 pandemic.

Another limitation was that the InStat Index does not consider the player’s positioning skills (movement and positioning without the ball), which is a vital part of a player’s performance. However, the InStat Index has been used as an outcome measure in a few published articles (Stanojevic and Gyarmati, 2016; Modric et al., 2019; Kubayi, 2020), and additionally shows a strong negative correlation between the mean team InStat Index and the actual position in the league at the end of the season. The InStat Index algorithm was altered after we collected the data, and scores in this article are therefore not compatible with present scores.

### 4.2. Conclusion

Although more research is required to further understand the nature of how psychological factors affect the performance of individual players, this study presents a very important result—namely that a perceived mastery motivational climate and extraversion are predictive of match performance. A mastery climate is created and modified by the coaches, and it is crucial that they are aware of how to create and maintain such a climate. This should be emphasized by the clubs when establishing a new team of coaches, or in the training of coaches *via* the various football federations. Extraversion was the only individual psychological factor that was predictive of individual football performance in women’s football. As personality traits are considered to be relatively stable psychological attributes, it is not recommended to utilize interventions or programs based on personality traits as a means of improving player performance.

## Data availability statement

The dataset presented in this article is not readily available due to privacy concerns. Requests to access the datasets should be directed to the corresponding author. Requests to access the datasets should be directed to susann.d.pettersen@uit.no.

## Ethics statement

Ethical review and approval was not required for the study on human participants in accordance with the local legislation and institutional requirements. Written informed consent from the participants' legal guardian/next of kin was not required to participate in this study in accordance with the national legislation and the institutional requirements.

## Author contributions

SP, MM, RK, BH, L-MPR, and FA contributed to conception and design of the study. BH and SP performed the statistical analysis. SP wrote the first draft of the manuscript. MM, RK, BH, L-MPR, and FA wrote sections of the manuscript. All authors contributed to manuscript revision, read, and approved the submitted version.

## Funding

The study was funded by Tromsø Research Foundation and UiT—the Arctic University of Norway.

## Conflict of interest

The authors declare that the research was conducted in the absence of any commercial or financial relationships that could be construed as a potential conflict of interest.

## Publisher’s note

All claims expressed in this article are solely those of the authors and do not necessarily represent those of their affiliated organizations, or those of the publisher, the editors and the reviewers. Any product that may be evaluated in this article, or claim that may be made by its manufacturer, is not guaranteed or endorsed by the publisher.
